# A Web-Based Program for Informal Caregivers of Persons With Alzheimer’s Disease: An Iterative User-Centered Design

**DOI:** 10.2196/resprot.3607

**Published:** 2014-09-15

**Authors:** Victoria Cristancho-Lacroix, Florence Moulin, Jérémy Wrobel, Bénédicte Batrancourt, Matthieu Plichart, Jocelyne De Rotrou, Inge Cantegreil-Kallen, Anne-Sophie Rigaud

**Affiliations:** ^1^Assistance Publique des Hôpitaux de Paris (APHP)Hôpital Broca, Service de GérontologiePôle de GériatrieParisFrance; ^2^Research team Equipe d'accueil 4468. Maladie d'Alzheimer, facteurs de risques, soins et accompagnement des patients et des famillesInstitut de PsychologieUniversité Paris DescartesParisFrance; ^3^Institut du Cerveau et de la Moelle épinière (ICM), Unité mixte de recherche (UMR) 975Institut national de la santé et de la recherche médicale (INSERM) U 1127, Centre national de la recherche scientifique (CNRS) UMR 7225Université Pierre et Marie CurieParisFrance; ^4^Inserm UMR-S970 - Centre de recherche cardiovasculaire de ParisEquipe 4: Epidémiologie cardiovasculaire et mort subiteParisFrance

**Keywords:** usability, caregivers, Alzheimer's disease, psychoeducational program, psychological stress inclusive design

## Abstract

**Background:**

Web-based programs have been developed for informal caregivers of people with Alzheimer’s disease (PWAD). However, these programs can prove difficult to adopt, especially for older people, who are less familiar with the Internet than other populations. Despite the fundamental role of usability testing in promoting caregivers’ correct use and adoption of these programs, to our knowledge, this is the first study describing this process before evaluating a program for caregivers of PWAD in a randomized clinical trial.

**Objective:**

The objective of the study was to describe the development process of a fully automated Web-based program for caregivers of PWAD, aiming to reduce caregivers’ stress, and based on the user-centered design approach.

**Methods:**

There were 49 participants (12 health care professionals, 6 caregivers, and 31 healthy older adults) that were involved in a double iterative design allowing for the adaptation of program content and for the enhancement of website usability. This process included three component parts: (1) project team workshops, (2) a proof of concept, and (3) two usability tests. The usability tests were based on a mixed methodology using behavioral analysis, semistructured interviews, and a usability questionnaire.

**Results:**

The user-centered design approach provided valuable guidelines to adapt the content and design of the program, and to improve website usability. The professionals, caregivers (mainly spouses), and older adults considered that our project met the needs of isolated caregivers. Participants underlined that contact between caregivers would be desirable. During usability observations, the mistakes of users were also due to ergonomics issues from Internet browsers and computer interfaces. Moreover, negative self-stereotyping was evidenced, when comparing interviews and results of behavioral analysis.

**Conclusions:**

Face-to-face psycho-educational programs may be used as a basis for Web-based programs. Nevertheless, a user-centered design approach involving targeted users (or their representatives) remains crucial for their correct use and adoption. For future user-centered design studies, we recommend to involve end-users from preconception stages, using a mixed research method in usability evaluations, and implementing pilot studies to evaluate acceptability and feasibility of programs.

## Introduction

### Background

Psycho-educational interventions have shown benefit in relieving the burden of caregivers of people with Alzheimer’s disease (PWAD), and associated manifestations of caregivers’ distress [1,2]. However, these programs are often implemented on-site in individual or group sessions, and may thus not be available for many caregivers who are overwhelmed or isolated, are unwilling to resort to available community help [3], live in remote regions [4], or are still in active life.

With the proliferation of information and communication technologies, there has been a growing interest in developing distance-based interventions that might be useful for this particular population of caregivers. Internet-based interventions have shown promising improvements in psychological [5-7], and physical outcomes [8]. Among these interventions, Web-based programs have shown to better respect the caregiver’s privacy and respond to availability issues than telephone-based interventions [9]. Moreover, the recent assessment report of the French Alzheimer's Plan 2013 [10] recommends the use of Web-based interventions in order to inform and support family caregivers.

### User-Centered Design Approach

However, one limitation of Web-based programs resides in the obstacles caregivers face adopting and making correct use of them [11]. The majority of caregivers of PWAD are over 65 years of age [12]. The typical changes accompanying aging (sensorial, perceptive, cognitive, and motor age-related declines), make it even more difficult for them to interact with technological systems [13]. Moreover, most of the older adults are also limited by their narrow experience with the Internet and by the lack of usability of some websites [14]. These aspects have been taken into consideration during the development process of our Web-based program.

In fact, the user-centered design approach fosters the conception of accessible products, and targets the needs of end users. Usability testing is a user-centered design method, which aims to identify the problems users are confronted with when using (technological) products, and to find the means of solving them [13]. To our knowledge, despite the benefit of usability testing in favoring the adoption and correct use of Web-based programs intended for caregivers of persons with dementia, few authors reported the use of this method or the adoption of a user-centered design approach in the development of their programs [6]. In contrast, usability studies are more frequent for programs targeting other populations, such as adolescents with overweight [15], or patients with chronic obstructive pulmonary disease [16]. To our knowledge, this is the first published work describing the user-centered design applied in the development of a program for caregivers of PWAD before it is tested in a randomized clinical trial.

### The Present Study

In fact, we aimed the application of user-centered design approach in developing a fully automated Web-based psycho-educational program called Diapason. This program was adapted from a face-to-face intervention, developed and tested by our team in order to reduce or prevent caregivers’ stress [17]. The Diapason program delivers: (1) disease information in twelve weekly sessions, (2) relaxation guidelines with training videos, (3) caregivers’ testimonials, and (4) stimulation activities for the relatives. This program is available in a free fully automated computerized and password-protected website. In this paper, we describe the iterative process that allowed for the adaptation of the program’s content and design.

## Methods

### Design

This was an exploratory-descriptive study, which consisted of a double iterative design allowing for the adaptation of the content and usability of the website. A group of health professionals (project team) participated in the iterations for determining the content, layout, and program design in the different stages of development through the workshops. In parallel, we conducted a proof of concept with caregivers and two usability tests with healthy older adults. The latter were based on a mixed research method with a convergent parallel design. Indeed, the protocol of the usability tests consisted of qualitative and quantitative data that were collected concurrently, but analyzed separately, and finally merged during the interpretation [18]. We used this method in order to obtain a more comprehensive analysis of data, and to raise the reliability of results. All the participants gave their written informed consent prior to their inclusion in the study.

### Diapason Program Development Process

#### Overview

**Figure 1:**
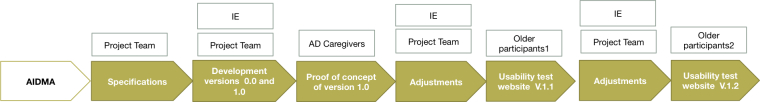
Face-to-face program, AIDMA=Aide dans la maladie d’Alzheimer, AD=Alzheimer’s disease, IE=Informatics engineer.

The program development process took place from 2009 to 2011 and included the following component parts: (1) design and development of the first two versions of the website, (2) proof of concept, and (3) two iterative usability tests (Figure 1 shows the development process).

#### Project Team Workshops

##### Participants

The project team comprised twelve health care professionals and researchers who participated in the regular meetings, 2 physicians, 8 psychologists, and 1 sociologist, all from the same geriatric department, as well as an informatics engineer.

##### Procedure

Throughout the whole development process, two psychologists (FM or VCL) moderated and conducted regular workshops in an informal setting with the project team. During each workshop, their specifications and recommendations were collected by one of the moderators. Based on their feedback, the informatics engineer built the website prototype (V.0.0), and its successive versions for the proof of concept and usability tests.

In addition, the project team analyzed the offline prototypes during the workshops. The analyses were focused on the following criteria inspired from usability guidelines [13,14,19,20]: (1) avoiding technical terminology (neither medical- nor informatics-related); (2) ameliorating accessibility for nonexperienced users, providing a familiar look (eg, looking like a printed notebook); (3) improving readability (including font size and contrast); (4) facilitating navigation (eg. providing visual cues); and (5) adapting the content to the target users (privileging condensed, clear, quick, and easily accessible information).

#### Proof of Concept

##### Participants

We recruited six informal caregivers of PWAD who attended the memory clinic, including three children, mean (SD) age 50.3 (12.4) years, and three spouses, mean (SD) age 73.4 (7.5) years, having at least once used the Internet. A purposive sampling approach was used to recruit the same number of children and spouses. Purposive sample techniques involve selecting certain units based on specific purposes rather than randomly. These techniques are used when the researcher wants to “set up a comparison between different types of cases”; it allowed us to compare the opinions of younger and older users about the program.

##### Procedure and Evaluation Tools

Each participant received a clear explanation of the program’s aim, and then browsed the Diapason website offline v1.0. After that, a semistructured in-person interview carried out by a psychologist (≈ 40 minutes) was recorded in order to evaluate the opinions of caregivers.  Semi structured interviews have a flexible and fluid structure, organized around an interview guide [21]. Questions regarding website usability and appearance were covered in the course of the interview.

#### First Usability Testing (Test 1)

##### Participants

As mentioned in the Introduction, since older people experience more difficulties with Internet use than other caregivers, we targeted them for usability testing. In order to avoid the learning bias, we recruited two different groups for each prototype version. There were 16 self-reportedly healthy persons 60 years and older (age mean 73.81, SD 7.03), having at least once used the Internet, that were recruited from three seniors associations in Paris. Sociodemographic information is summarized in Table 1.

**Table 1:** Sociodemographics of usability test participants.

Characteristics		Test 1 mean (SD) or n (%)	Test 2 mean (SD) or n (%)
**Participants gender**			
	Male	4/16 (25)	2/15 (13)
	Female	12/16 (75)	13/15 (87)
Participants age (years)		73.81 (7.03)	72.12 (7.03)
Internet experience (years)		8.32 (6.79)	8.91 (8.07)
Frequency of Internet use (days per month)		25.31 (10.22)	22.33 (10.32)

##### Procedure and Evaluation Tools

**Figure 2:**
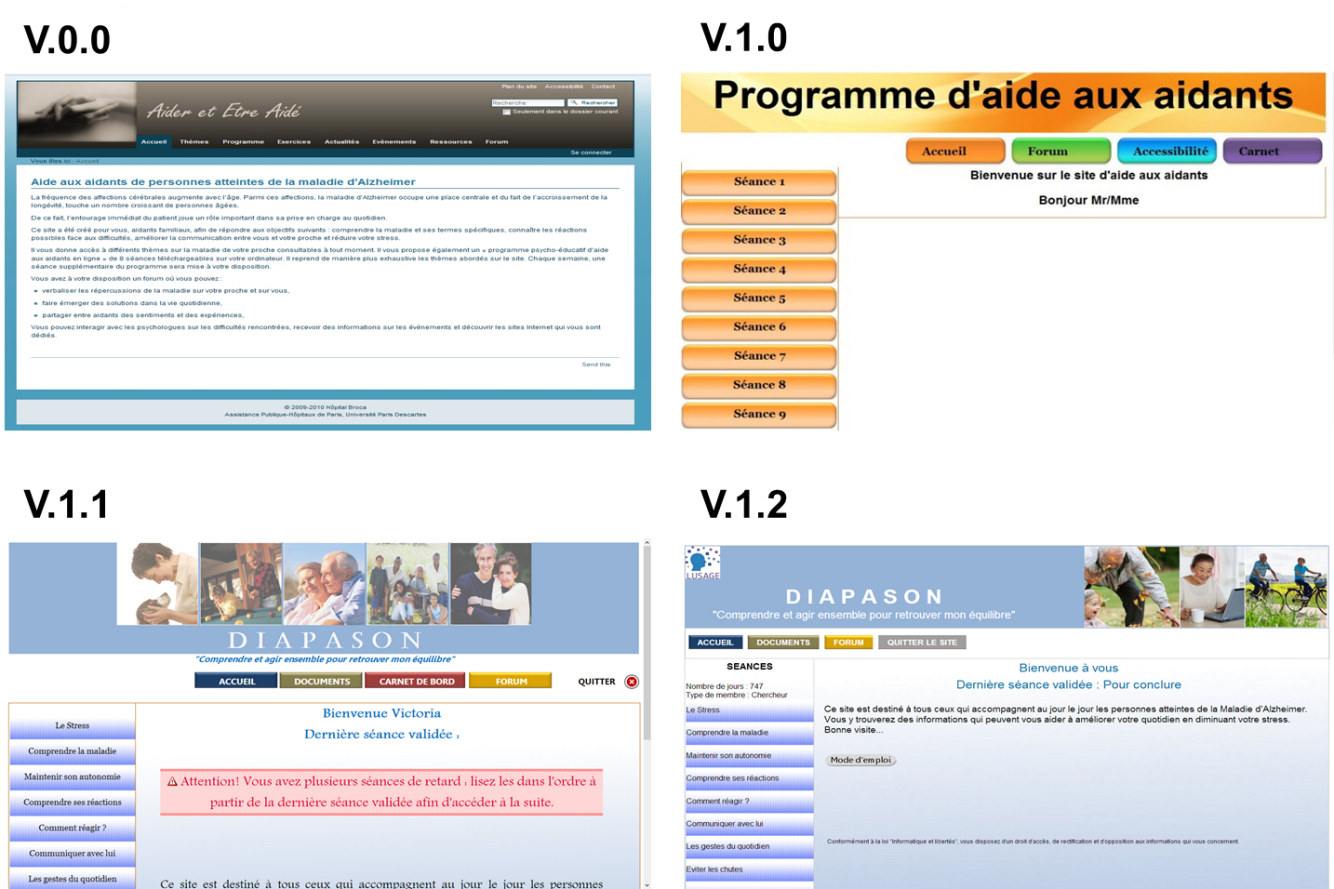
Diapason website program versions.

A research psychologist conducted a one hour individual usability test with each participant. The session was divided into four steps:

1. The participant filled out a questionnaire on sociodemographic data, Internet experience, and the monthly frequency of Internet use.
2. The participant was asked to follow written instructions (Textbox 1) of navigation on the offline version of v1.1 (Figure 2 shows this version) using a “think aloud” method [22]. In the “think aloud” method, which is common in usability testing, the users are asked to think aloud while using the system, allowing the evaluator to understand what they are doing and the reasons for their actions [13]. The five tasks were selected to cover the main functions of the website. The test sessions were video-recorded for a behavioral analysis. Moreover the psychologist noted the participant’s mistakes, difficulties or comments, and avoided to interfere with the evaluation.
3. The participant’s opinions of website usability were assessed with a five point Likert scale (0= negative to 4= positive) designed by our team (VCL). The survey evaluated five topics: (1) overall impression about the website; (2) easy-to-use perception; (3) pleasant to use perception; (4) coherence of website layout; and (5) satisfaction with the website design (font, colors).
4. At the end, the participant was asked to answer a semistructured interview on the following topics: (1) positive and negative aspects of the website, (2) difficulties when using the website, (3) discomforting situations during navigation on the website, and (4) advice to improve the appearance and design.

**Five step usability test.**

1. Please enter to the website: www.etreaudiapason.com
  - Username: *Participant*
  - Code: 123456
  - Go back to the home page
2. Go to the session “*Managing the caregivers stress*”
  - Watch the video “*caregiving-related stress*” and change to full-screen
  - Go back to the home page
3. Search the glossary
  - Read the meaning of the word “*hippocampus*”
  - Go back to the home page
4. Go to the stories
  - Read the story of “*Lucia*”
  - Go back to the home page
5. Go to the forum
  - Post the message: *I’m using Diapason*
  - Go back to the home page

#### Second Usability Testing (Test 2)

##### Participants

We recruited 15 healthy volunteers over 60 years old, age mean 72.12; SD 7.03, through three seniors associations in Paris. They had at least once used the Internet. Sociodemographic information is summarized in Table 1.

##### Procedure and Evaluation Tools

With the second usability test, we evaluated the offline v1.2 (Figure 2). The protocol was identical to the first usability test.

### Analysis Methods

Qualitative data from the workshops (ie, moderator’s notes), the proof of concept (ie, interviews), and the usability tests (ie, interviews and mistakes, difficulties or comments; observed and collected by the evaluator) were analyzed based on the thematic analysis method [23]. After being familiarized with data JW and VCL coded the relevant extracts of material concurrently. Then, they analyzed the themes based on the recommendations of various usability authors [13,14,24,25]. Finally, they corroborated the pertinence of the selected topics, comparing them with initial verbatim.

Assisted by the software “The Observer XT” and an observation grid, two trained psychologists (VCL and JW) collected, coded, and analyzed videos of usability tests. We measured the frequency of mistakes, requests for help, and the duration of task performance.

Finally, we analyzed the satisfaction survey results using descriptive statistics.

## Results

### Participants

In total, 49 persons were involved in the Diapason program development: 12 health care professionals, 6 caregivers, and 31 healthy older adults. The development process resulted in four successive website versions as shown in Figure 2 for which the qualitative results of the iterative design are provided in Table 2.

### Qualitative Results

#### Project Team Workshops

The results of the first workshops showed that most professionals were motivated by the new project. They proposed interesting and creative ideas to develop the Web-based program. They stated that the Diapason program should be made easily and rapidly accessible to overwhelmed caregivers who may have only fifteen or twenty minutes to spend with the program. Some professionals also expressed concerns about the suitability of Internet use for caregivers, since most of them were spouses of patients and likely inexperienced with this technology. Some also thought that computers might increase caregivers’ isolation.

**Figure 3:**
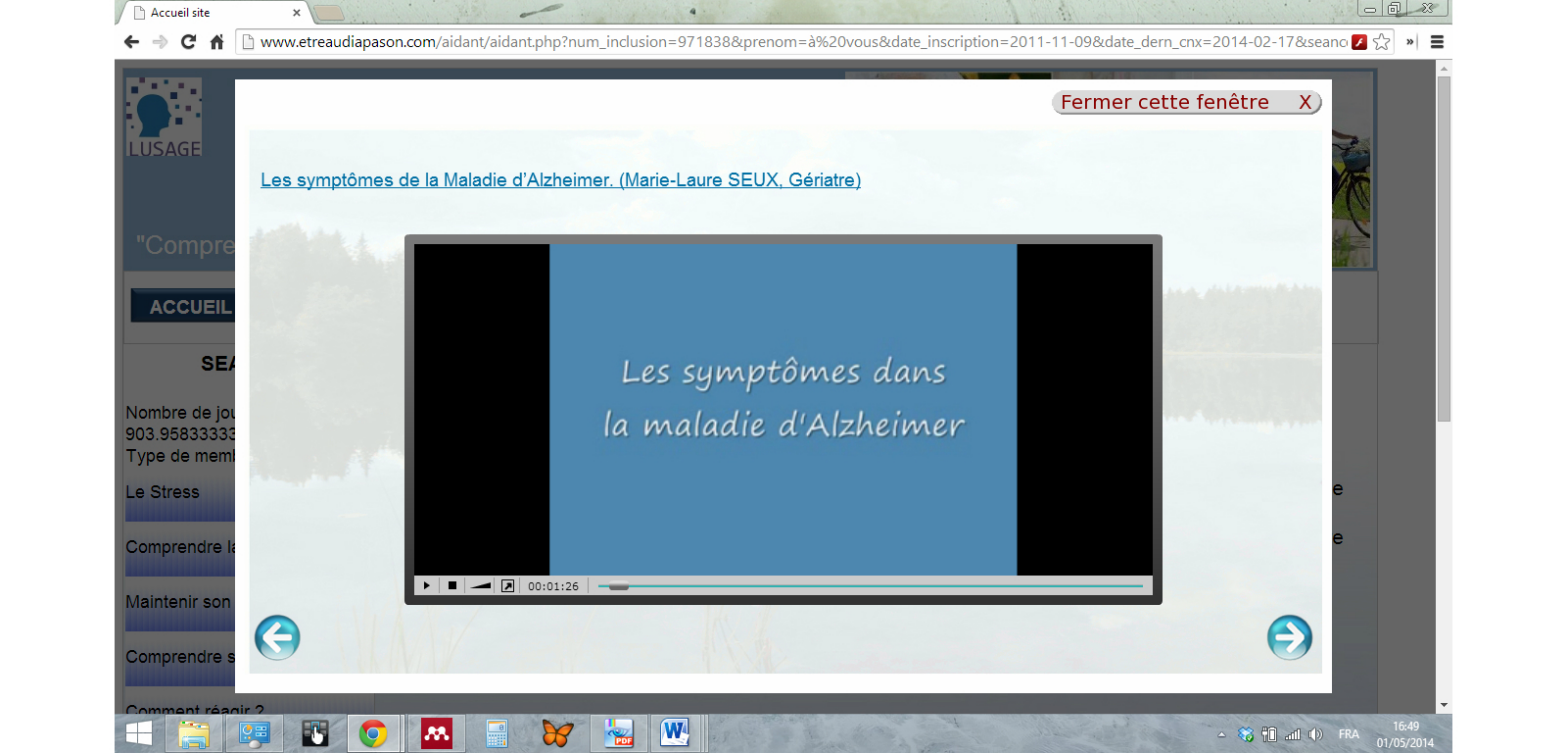
Screen display in the Diapason website.

Based on the criteria selected by the team (described above in the Procedure of Workshops), the website v0.0 was not retained. The content was too long, complex, and technical for nonprofessionals. The appearance was dark, sad, and stigmatizing (Figure 2). As for v1.0, the project team suggested the use of a more “common” language for the button sections. They also recommended using a “light box” effect, to facilitate navigation (Figure 3 shows this display). Concerning v1.1**,** the team found “My journey” functionality unnecessary or infeasible. It was also suggested to add a “Relaxation training” in the program. As regards v1.2, the professionals supervised the consistency of changes made by the informatics engineer on the website following the demands of end-users, and prioritized them, based on their feasibility and relevance.

**Table 2:** Qualitative results.

Website version		Category	Problem reported	Actions and/or solutions
**PT** ^a^ ** workshops**				
	0.0	Readability	Content too complex, using technical jargon	Contents were simplified, avoiding medical or informatics jargon
			Content too long, adapted for professionals caregivers	Contents and the layout were reedited
		Appearance	Black and gray colors, photo suggestive of sadness	The website was redesigned with “flashy” colors
**Proof of concept and PT** ^a^ **workshops**				
	1.0	Readability	Low contrast between characters and background of some website pages	The color of website background was modified
			Font size too small (12 point)	The font size was increased (16 point)
			Unfamiliar terminology	The terms were replaced, eg, “resources” by ”document”, “search engine” by “glossary”, “me/he/she” by “life’s testimony”, among others
		Ergonomics	Complex actions to access the “sessions”	Action was simplified
**Usability testing #1 and PT** ^a^ **workshops**				
	1.1	Ergonomics	Participants clicked twice on the hyperlink, but flash screen closed with the second click	Explanation in the Internet and printed user manual
			Lack of an icon to close the flash screen	Add the icon “close this window”
			Small characters at the forum section	[no quick solution]
			“Send the message” option is at the bottom of the website, and requires use of the vertical scrollbar	[no quick solution]
			Some participants are not familiar with video-player icons	Explanation in the Internet and printed user manual
**Usability testing #2 and PT** ^a^ **workshops**				
	1.2	Ergonomics	Dimensions of the website vary depending on Internet browser and computer models	[no quick solution]
		Navigation	Hyperlinks text was unfamiliar for some of participants	Explanation on the Internet and in a printed user manual

^a^ PT = project team

#### Proof of Concept

##### Overall Opinion of Caregivers

The caregivers found the website prototype (v1.0) clear and understandable. All of them, and especially the PWAD’s spouses, appreciated the aims, the topics, and the website's layout. Although the participants thought the Web-based program likely to be useful for isolated caregivers, most of them underlined the need to communicate with professionals and to maintain face-to-face contact.

The suggestions to change the look (adding photos and modifying colors) were implemented in the following version (v1.1). Moreover, the caregivers pointed out important usability issues.

##### Unfamiliar Terminology

**Figure 4:**
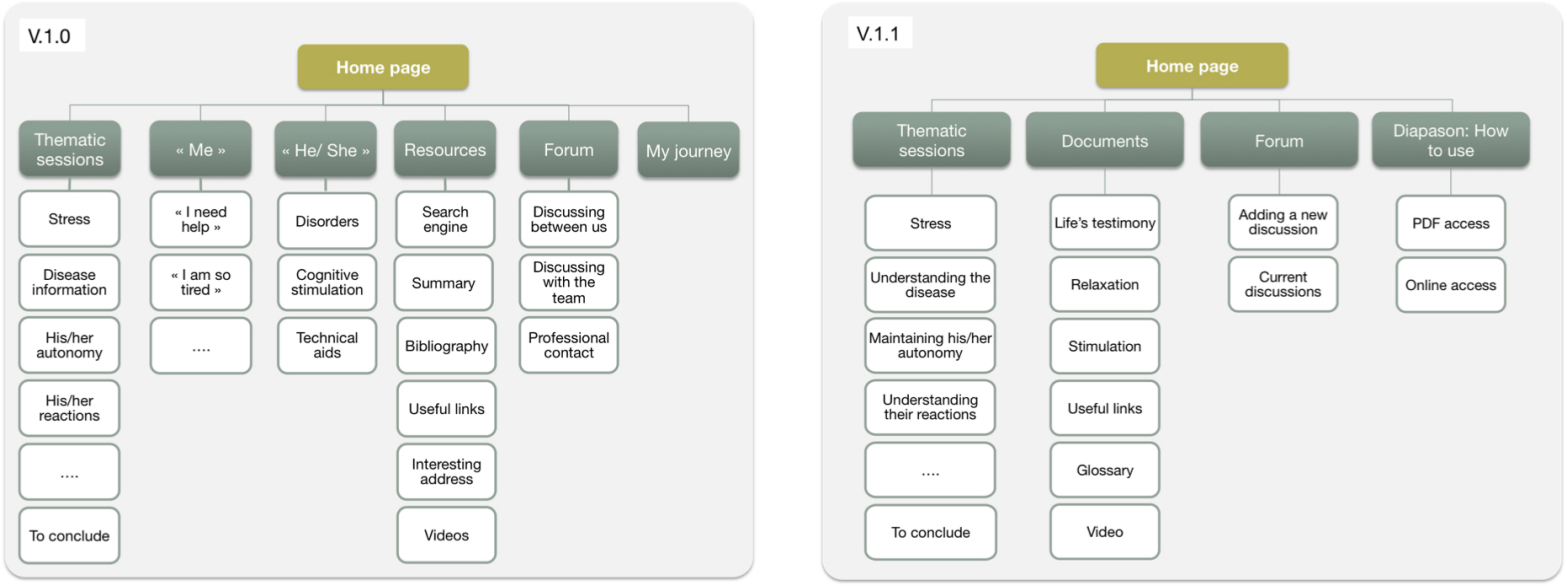
Layout for versions 1.0 and 1.1 - PDF=Portable document forwards.

Although the project team aimed to avoid jargon (from informatics or medical areas), some of the terms used remained confusing for the participants in this version. For instance, the “Resources” button (ie, “Ressources” in French), giving access to additional sections (eg, relaxation training, glossary, etc), was understood as giving access to financial help. Consequently, the website was reorganized, and potentially confusing words or expressions were replaced by more commonly used website terminology (Figure 4 shows this layout).

##### Font Readability

The younger caregivers found the font size too small and thought it would constitute an obstacle for older users. By contrast, older participants did not raise this issue, but reported that some pages were difficult to read due to the lack of contrast between the background and the font. In the subsequent version (v1.1) the font size was increased and the contrast enhanced.

##### Ergonomics

To access the sessions, the user had to click on a button, and then confirm their choice by clicking on another one. This condition was simplified.

##### Simplified Layout

Based on the project team and on caregivers’ suggestions, the website’s layout was simplified (Figure 3). The version v1.1 and the final version only offered three main sections: (1) thematic “sessions”, (2) a “forum”, and (3) the “documents” providing access to other content (eg, relaxation or glossary).

Unfortunately the option “contact a professional” and the videoconferencing options were not implemented, owing to a lack of resources. Moreover, the section “My journey”, a private diary for caregivers, was removed because the system could not encrypt the data.

#### Usability Test of v1.1

In this version, the overall program content was added to the website. Moreover, the readability improvements performed during the proof of concept phase were appropriate, as no participant reported any visual discomfort while browsing the website (except for the forum, as described in this section). Concerning the easy-to-learn perception, many participants asserted that they would have performed better if they had used the website more than once.

Using the website would be easy provided I received training or that I spend more time using it.

Mrs. H, 71 y/o

However, various ergonomics issues were identified. Although the website home page was kept accessible using a script (JQuery Superbox) to display a screen with a light box effect (Figure 3), most participants did not know how to go back to the previous page.

To go back to the home page sometimes I had to click in a grey zone or sometimes click on the close button, this is not practical.

Mme GG, 69 y/o

To correct this, we added an icon at the top right of the screen with the message “Close this window” (in Figure 3, the button “*Fermer cette fenêtre*”). Some other issues remained unsolved due to technical or logistical reasons: (1) the font size and symbols in the forum and video-player interfaces did not facilitate reading; (2) the post button for forum messages was at the bottom of the screen, requiring the use of the scrollbar; and (3) the least experienced participants often double clicked in the website, which was in conflict with the one-click activated “light box” effect, as the second click immediately closed the window. For each of these problems, a clear explanation was provided in the user's manual.

#### Usability Test of v1.2

There were two additional problems that were identified during the second usability test. The website display varied according to the Internet browser and/or the computer model, and some participants did not know how to use the hyperlinks in the website. We adapted the Internet and printable version of the user’s manual, taking into account the results of both usability tests, including the issues without a quick or easy solution (Table 2).

#### Additional Findings From the Usability Tests

Although during the usability tests the evaluators found most of the problems reported by users in the interviews, the evaluators also identified additional problems regarding the computer interface, and the Internet browsers. The mouse cursor and the scrollbar were not visible enough on the screen (lack of contrast or small size), and some participants did not distinguish the website settings from the Internet browser or computer interface. For instance, a participant recommended changing the order of icons of the Internet browser because he thought that the latter was part of the website. When asked to go back to the “home page” of the website, another participant closed the browser window, then could not find, unaided, the icon of the Internet browser to continue the task. These problems were observed even for the people with more than one year of experience of Internet use.

### Quantitative Results of Usability Tests

#### Behavioral Analysis of v1.1 and v1.2

There were two psychologists using an observation grid who analyzed the videos of usability tests sessions with The Observer TX. The three main variables analyzed are presented in Table 3: (1) the duration of the task, (2) the total of errors, and (3) requests for help during the evaluation. We observed an important reduction in completion time and the total of requests for help after the website improvements were made between the first and the second iteration. However, the overall number of errors remained similar in the two versions, possibly owing to unsolved usability problems.

**Table 3:** Total performance in five step usability test (for v1.1 and v1.2).

Usability tests	Mean task completion time, seconds	Total group errors (n*error)^a^	Total group requests for help (n*help)^b^
v1.1, n=16	1866.14	103 (15)	36 (6)
v1.2, n=15	1042.40	96 (15)	5 (4)

^a^ n*error, number of persons who made at least one error

^b^ n*help, number of persons who asked for help

#### Usability/Satisfaction Survey

As shown in Table 4, the two website versions yielded similar scores. Overall the participants’ opinions of the website were positive. The lowest scores were for the system’s “ease of use”. A plausible explanation was that most of the participants evaluated website ease of use for themselves, but not for other seniors. During the semistructured interviews, the most prominent argument was that the “other seniors” might be in poorer health and cognitive status than the participant himself. This suggests that this item reflects the participants’ perception of older adults more than their experience using the website.

**Table 4:** Results of the usability/satisfaction 5 Likert questionnaire.

Satisfaction questionnaire items	Version 1.1 mean (SD)	Version 1.2 mean (SD)
Overall website evaluation	3.19 (0.54)	2.80 (0.68)
Easy-to-use	2.75 (0.68)	2.47 (1.06)
Pleasant to use	2.94 (0.68)	2.60 (0.63)
Website structure	3.31 (0.70)	3.13 (0.83)
Website layout	3.07 (0.70)	3.00 (0.76)
Website font	3.19 (1.05)	2.87 (1.06)
Website colors	3.19 (1.28)	3.67 (0.40)
Overall mean score	21.63 (2.90)	20.53 (3.40)

## Discussion

### Program Development

In this paper, we describe the iterative development of a Web-based psycho-educational program (Diapason) aiming to reduce or prevent stress in caregivers of PWAD. To our knowledge, this is the first published work describing a user-centered design process for the development of a program addressed to caregivers of PWAD. To that end, we involved end-users and health care professionals in a double iterative design, allowing for a cyclic adaptation of the content and design to the targeted population. During the whole process, our project team elaborated tailored guidelines for the engineer’s mission, based on their own professional experience, but also taking into accounts the feedback from end users.

In fact, the involvement of end-users was decisive in the development of our program. The caregivers and healthy older adults pointed out various website usability deficiencies which had been unnoticed by the professionals. In agreement with the user-centered design approach, our aim was to prevent users lacking the necessary cognitive (experience or abilities) or physical resources from having to deal with the maladjusted and imposed technology devices [13]. Various authors have demonstrated the relevance of this approach to design eHealth interventions. For instance, Chiu and Eysenbach [11] found that caregivers attracted to a service which they considered useful, could eventually stop using it if they perceived the service as nonuser-friendly. Furthermore, focusing on caregivers’ needs (and their representatives) during the development process is a critical aspect for the acceptability and adoption of interventions [6].

### Principal Findings

The Proof of Concept evaluated the program's content and website usability, and was carried out with a group of caregivers of PWAD, consisting of children and spouses. As hypothesized by our team, and in accordance with the literature [13], the difficulties linked to usability issues were preeminent in older participants. Thus, the usability tests were focused on adapting the program in a senior-friendly website. As a consequence, we decided to privilege the recruitment of a group of healthy older persons rather than the (overwhelmed) caregivers for usability tests.

In order to obtain a more comprehensive appraisal of usability tests’ results, we designed a mixed research method combining behavioral analysis with think aloud method, individual interviews, and questionnaires [13]. In this study, the questionnaire was the least sensitive and informative of the three methods. A plausible explanation is that closed-ended questions offer answers on “what” the users' opinions are, or “how” difficult the website is to use, but they do not give information as to “why” this might be. For example, researchers may obtain information on the degree of disagreement about an item, but not “why” the subject disagrees with it. In contrast, interviews and behavioral analysis (using thinking aloud) provided us with valuable and accurate data about the difficulties that users encountered in the website. For instance, additionally to usability issues described elsewhere, we observed that the participants confused the website and the Internet browser interfaces, and some had many difficulties with the computer interface or Internet browser themselves. As stated by Nielsen, even the most recent and popular operating systems Interfaces could present important usability issues, which entail cognitive overhead and add memory load [26]. Therefore, designers and evaluators of website usability should effectively disentangle website conception issues from problems due to computer and Internet environments (eg, Windows 8, Internet browser...).

It is also noteworthy that most of the older adults who filled out the satisfaction questionnaire during usability tests considered the website easy-to-use for them, but not for other seniors. They argued that they thought about older adults with poorer health and more perceptual and cognitive deficits than themselves. This result matched those of previous studies by our team [27]. In both projects we explained to the participants (older adults) that the study aimed to identify their needs to create a senior-friendly technology. As described in this study, older adults rarely identified themselves as the “target” of gerontechnology, which was not intended for them, but for “other” older adults who may be (much) older, frailer, and more isolated than they are. This attitude may be due to “negative self-stereotyping”, described in the literature [28,29]. In our study, children of PWAD (see in Proof of Concept section) also expressed this stereotyping of aging people. These results prove the advantage of observation methods, which provide an objective basis for the (un)necessary improvements.

As regards the program’s content, the project team designed the Web-based Diapason program based on the Aide dans la Maladie d’Alzheimer (AIDMA) program content, retaining the most pertinent information and making it more accessible and easier to use. In fact, the AIDMA program was proposed in 2 hour face-to-face sessions, while we adapted the Web-based program to be used 15-20 minutes per week. Nevertheless, slight changes in topics were required through the development process, since some of them had already been tested by our team in the AIDMA project [17], and improved based on professionals’ and caregivers’ feedback.

Finally, although most of the professionals and end-users judged the Web-based program likely to be useful for isolated caregivers, some of them also worried that these interventions might increase (or reinforce) caregivers’ isolation. We also encountered health care professionals who rule out the use of technologies and claim face-to-face interventions are the only way to help patients and their families. In our team, even if we recommend the use of face-to-face interventions, we also consider it appropriate to propose additional support for caregivers or for patients who cannot benefit from on-site psycho-educational programs.

### Limitations and Lessons Learned

The acknowledged limitations of the present work might be useful for methodological and logistic considerations in future projects. First, even if our usability questionnaire was more adapted to our project context, it did not include items intended to measure “learnability” and “usefulness” perceptions [30], instead, we conducted the interviews at the end of the evaluations exploring these constructs. To improve the analysis of both dimensions, we recommend to conduct a field study during the development process, such as pilot tests in which the users have access to the program for one or two weeks [31]. In fact, these two measures would be valuable if some usability issues remained unsolved, as in our study. The difficulties encountered by the users may demand a learning process, and the developer has to know whether the website facilitates this process. Additionally, a pilot test may be a reassuring step before a clinical trial.

In this work, the involvement of both professionals and end-users was critical to develop a ready-to-use eHealth program [13,14,32]. Moreover, this work provides additional arguments supporting the effectiveness of using usability guidelines to increase Internet accessibility for older adults [20]. Nonetheless, based on our current knowledge about website usability for seniors, we think that some ergonomic mistakes in the first versions of our website could have been prevented earlier in the development process, with the help of an expert in ergonomics at these stages. For instance, we recommend avoiding the use of open source “ready-to-use” programs, since they do not always respond to the universal design criteria.

Finally, and in accordance with other studies [20], our findings highlighted the relevance of using a mixed method approach, combining subjective and objective methods, such as observation analysis and interviews, to obtain complementary data.

### Conclusions

The implementation of Web-based programs requires the adaptation of the system, including content and ergonomics, to match the needs of target populations. In fact, even when the content and aims are well established and tested, the face-to-face programs need to be reviewed and adapted for Internet use. Only the optimal usability and readability of interventions may prevent the underuse or incorrect use of these programs. Through usability iterative evaluations, the latest website version of our program has been improved, and is currently being tested in a randomized clinical trial [33]. For future user-centered design studies we recommend the following: (1) involving end-users from preconception stages, (2) using a mixed research method (mainly based on interviews and observations) in usability evaluations, and (3) implementing pilot studies to evaluate acceptability and feasibility before a clinical trial.

## Abbreviations

AIDMA: Aide dans la Maladie d’Alzheimer
PWAD: people with Alzheimer’s disease
